# Radiosurgery for Epidermoid Tumors: Dramatic Pain Relief from Trigeminal Neuralgia

**DOI:** 10.7759/cureus.6448

**Published:** 2019-12-23

**Authors:** Yoshihisa Kida, Yoshimasa Mori

**Affiliations:** 1 Neurosurgery, Ookuma Hospital, Nagoya, JPN; 2 Radiation Oncology and Neurosurgery, Shin-Yurigaoka General Hospital, Kawasaki, JPN

**Keywords:** epidermoid, facial spasm, gamma knife, radiosurgery, trigeminal neuralgia

## Abstract

Purpose

The purpose of this study is to discuss the long-term effects of radiosurgery for epidermoid tumors, including the symptoms of trigeminal neuralgia and/or facial spasm, which we have originally reported before.

Background

Intracranial epidermoids are slow-growing tumors that can become symptomatic once they develop into large tumors. The mainstay of the treatment is surgery. However, eradicating the whole tumor is often difficult and some tumors may recur. In addition to their mass effects on the brain, these tumors are often associated with hyperactive nerve dysfunction syndromes such as trigeminal neuralgia, glossopharyngeal neuralgia, and/or facial spasm.

Cases and methods

We report 13 cases of epidermoid tumors, 12 of which were located in the cerebellopontine angle (CPA), which were treated using 14 radiosurgery procedures. The inclusion criteria for radiosurgery were the presence of well-localized small tumors and/or severe associated neuralgia or facial spasms. The mean target volume ranged from 0.17 to 9.5 cm^3^ with a mean of 2.85 cm^3^. The lesions were treated with a mean maximum and a marginal dose of 28.2 Gy and 14.2 Gy, respectively.

Results

Among the 14 gamma knife procedures that were performed in 13 patients, dose planning to ensure total and partial coverage for relief from hyperactive cranial nerve dysfunction (HCND) was performed. Six cases were totally and another eight were partially covered at the dose planning. The irradiated tumors showed a minor decrease or no remarkable changes during a mean follow-up period of 86.1 months. Tumor progression requiring a second surgery was seen in two cases. The trigeminal neuralgias either improved or disappeared soon after the procedure, enabling the discontinuation of the medication.

Conclusion

Radiosurgery led to a dramatic improvement in HCND. In fact, the immediate cure of neuralgia after the radiosurgery was observed in several cases, even after partial coverage with radiosurgery. The interface between the tumor and the nerve was the main target. The definite mechanisms for this favorable outcome have not been verified yet, but the functional modulation by the radiosurgery could be one. Electrophysiological alteration at the interface between the tumor and nerve has been considered. When the tumors were totally covered with radiosurgery, persistent tumor control was expected. Sufficient tumor control is possible if the tumor can be covered entirely with radiosurgery. Functional modulation of trigeminal neuralgia and facial spasms can also be attained even by partial dose planning for the nerve-tumor interface.

## Introduction

Epidermoid tumors are generally slow-growing tumors. They are thought to develop between the third and fifth weeks of gestation from ectodermal remnants during neural tube formation in embryogenesis. Intracranial epidermoid tumors account for 1-2 % of the reported intracranial tumors [1]. These tumors may be found chiefly in and around the cerebellopontine angle (CPA) or parasellar region. Since the tumors grow very slowly or are often indolent, they have typically developed into moderately large tumors by the time of their discovery. They become symptomatic by compressing the surrounding brainstem or stimulating near-by cranial nerves. 

Besides the mass effects of brain compression caused by these tumors, epidermoids often cause hyperactive cranial nerve dysfunction (HCND) such as trigeminal or glossopharyngeal neuralgias or facial spasms [2,3]. Cranial nerve involvements often require adequate and prompt resolution to relieve these troublesome symptoms. Chemical meningitis is another special manifestation of this tumor, which is caused by the possible leakage of accumulated keratin and cholesterol inside the cyst into the subarachnoid space. 

The mainstay of current treatments is surgical resection, but total resection of the tumors is often difficult and often hazardous because of the possible damages to the brain or cranial nerves, especially when the tumor capsule is firmly attached to cranial nerves or the brainstem [4,5]. Since our own first report on the treatment of epidermoid tumors using radiosurgery, several other clinical reports have been presented in which the successful resolution of HCND has been emphasized [6,7-10]. Here, we discuss the long-term clinical results of epidermoid tumors treated with radiosurgery. The purview of our report extends beyond tumor control and consider functional outcomes such as trigeminal neuralgia as well [11].

## Materials and methods

Case selection

Epidermoid tumors usually develop into large tumors that extend widely into the cerebral cisterns or into the cistern on the opposite side. Consequently, treatment with radiosurgery is often difficult and surgical resection is usually the preferred treatment method. Only patients with small well-localized tumors with a mean dimeter of less than 30 mm are considered suitable candidates for radiosurgery with full coverage of the tumor volume. In addition, patients suffering from HCND such as severe trigeminal or glossopharyngeal neuralgias or facial spasms and large epidermoid tumors in CPA are sometimes considered for radiosurgery, with the intention of pain relief and partial coverage of the nerve and tumor in the cistern. 

**Figure 1:**
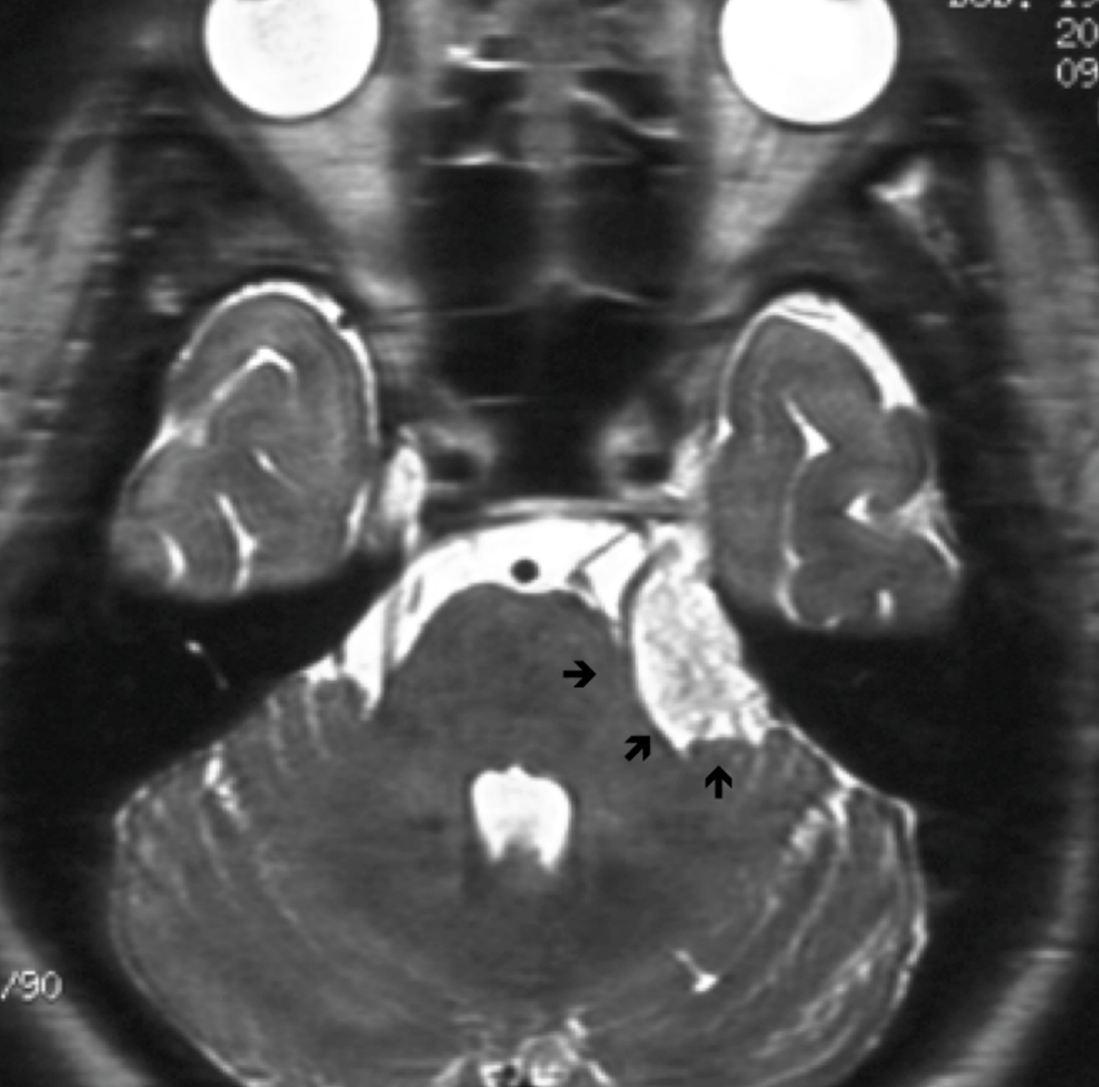
Characteristics of cases treated with gamma knife surgery Most of the tumors were located exclusively in or around cerebellopontine angle (arrows)

Here, we report 13 cases of epidermoid tumors treated with 14 radiosurgery procedures (two radiosurgical procedures were required in one case). There were six males and seven females, and these epidermoid tumors accounted for less than 1% of the brain tumors encountered during 20 years of the study period at our institute. The patients’ ages ranged from 7 to 80 years with a mean age of 47.2 years. The results of 14 trials of radiosurgery were summarized. Patients were suffering from trigeminal neuralgia in nine cases and/or facial spasm in two. HCND was recorded as the main neurological sign or as a single symptom (Table 1). In fact, many of the patients had either trigeminal neuralgia or facial spasms, at the same time or at different times during their clinical course. Other neurological deficits such as ataxia, diplopia, or hearing loss were also recorded. The locations of the lesions were exclusively within the CPA in 12 cases and within the interhemispheric cistern and pineal gland in one case each. Seven cases underwent surgical resection before radiosurgery to remove tumors and to obtain a histological diagnosis. The other seven cases were only diagnosed based on radiological studies such as CT scan or MRI. CT images showed a deep low-density spot while MRI showed an iso-intensity on T1 images and a high signal intensity on T2 images. First imaging steady-state acquisition (FIESTA) or heavy-T2 studies produced unique and specific images in the cerebral cisterns as shown in Figure 1.

**Table 1:** Characteristics of cases treated with gamma knife surgery M: male; F: female; CPA: cerebellopontine angle

No	Age (years)	Sex	Symptom	Location
1	42	M	Facial spasm	CPA
2	45	M	Trigeminal neuralgia	CPA
3	39	F	Trigeminal neuralgia	CPA
4	23	F	Diplopia	Pineal
5	43	F	Trigeminal neuralgia	CPA
6	46	M	Trigeminal neuralgia	CPA
7	65	M	Trigeminal neuralgia	CPA
8	80	F	Trigeminal neuralgia	CPA
9	7	F	Hearing loss	CPA
10	53	M	None	Frontal base
11	30	F	Trigeminal neuralgia	CPA
12	66	M	Trigeminal neuralgia	CPA
13	53	M	Trigeminal neuralgia	CPA
14	69	F	Facial spasm	CPA
Mean and total	47.2	M: 7; F: 7	Trigeminal neuralgia: 9; facial spasm: 2	CPA: 12; frontal: 1; pineal: 1

 

Radiosurgery

**Figure 2:**
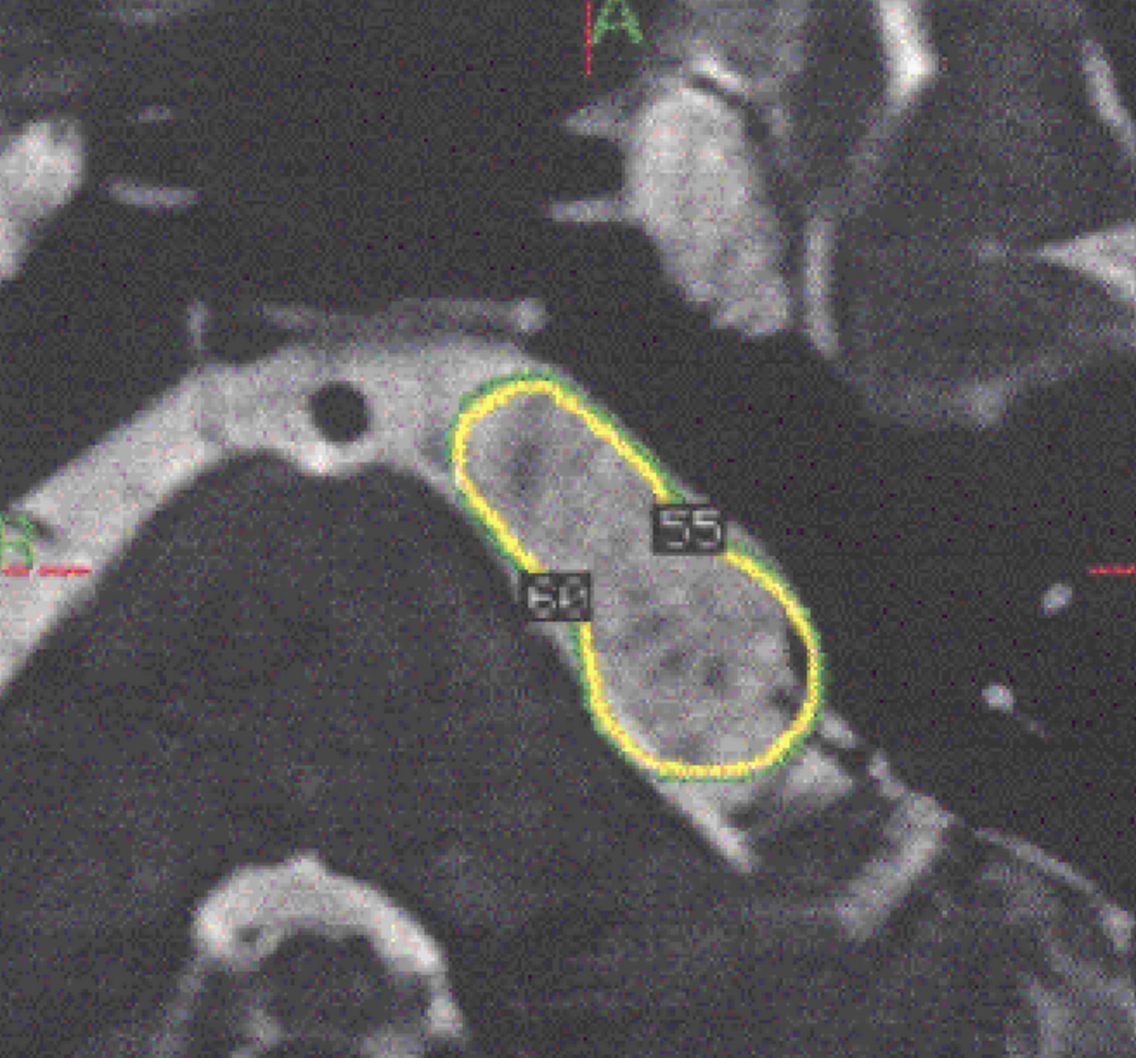
Whole tumor coverage at the radiosurgery Yellow line indicates the tumor margin and the target volume

Gamma knife treatment was performed under local anesthesia supplemented with intravenous anesthesias, and a Leksell head frame was used to stabilize the head of each patient. Enhanced T1 and T2 MRI images were obtained for dose planning with GammaPlan (ELEKTA, Stockholm). Coregistered FIESTA or heavy T2 studies were mainly used for dose planning to visualize all the tumor extensions into the cisterns. Two methods of radiosurgery were chosen. If the tumor was not large and well localized, the whole tumor was covered with radiosurgery (Figure 2).

**Figure 3:**
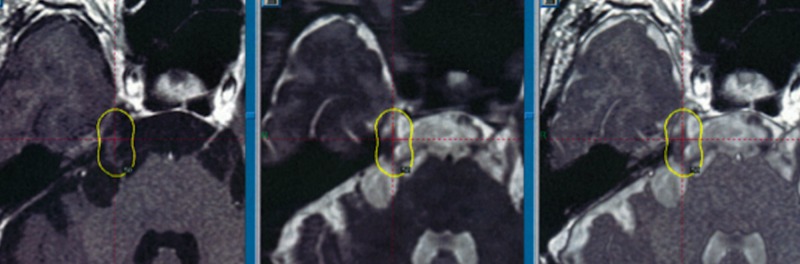
Partial tumor coverage

If, however, the tumor extended widely into the surrounding cisterns, only parts of the tumor that were circumscribed and had adhered to near-by cranial nerves in the cisterns were targeted with the intention of alleviating HCND symptoms such as trigeminal neuralgia and facial spasms (Figure 3). Dose planning was performed using GammaPlan (ELEKTA, Stockholm).

Follow-up studies

Medical follow-up studies consisting of neurological status assessments as well as imaging studies with enhanced MRI were performed every three months during the first year, and every six months thereafter. Trigeminal neuralgias are estimated with both a visual analog scale and demonstrated with a percentage decrease or increase of neuralgia with self-assessment by the patient and visual analog scale. Pain before and after gamma knife surgery was also evaluated retrospectively based on medical records using the Barrow Neurological Institute (BNI) pain intensity scale and the medication requirements. 

Statistical analyses

Statistical analyses were performed using the t-test for the comparisons of paired data between the treatments with whole or partial coverage. A p-value of less than 0.05 was considered to be statistically significant. 

## Results

The tumors, which had a mean volume of 2.85 cc, were treated using either whole (seven cases) or partial tumor coverage (seven cases) with a mean marginal dose of 13.1 Gy. The majority of tumors showed no apparent volume changes, however, some tumors demonstrated shrinkage. The improvements in HCND were remarkable even without medication. The details of the treatment with radiosurgery are summarized in Table 2.

**Table 2:** Radiosurgery for epidermoid tumors and clinical follow-up results CPA: cerebellopontine angle; Max D: maximum dose; Marg D: marginal dose; PR: partial remission; NC: no change; PG: progression

Neurological Signs	Location	Tumor (cm^3^)	Max D (Gy)	Marg D (Gy)	Coverage	Tumor response
1. Facial spasm	CPA	1.09	28	14	Partial	PG
2. Trigeminal neuralgia	CPA	0.52	24	14.6	Partial	PG
3. Trigeminal neuralgia	CPA	2.29	30	15	Partial	NC
4. Diplopia	CPA	9.5	26	13	Partial	NC
5. Trigeminal neuralgia	CPA	5.78	21	12.6	Whole	PR
6. Trigeminal neuralgia	CPA	1.5	28	14	Partial	NC
7. Trigeminal neuralgia	CPA	1.09	28	14	Partial	NC
8. Trigeminal neuralgia	CPA	2.51	28	14	Whole	NC
9. Hearing loss	CPA	1.13	23	16.1	Whole	PR
10. None	Interhemispheric cistern	3.7	28	14	Whole	PR
11. Trigeminal neuralgia	CPA	1.64	28	14	Partial	NC
12. Trigeminal neuralgia	CPA	0.82	23.3	14	Whole	NC
13. Trigeminal neuralgia	CPA	6.48	25	12.5	Whole	PR
14. Facial spasm	CPA	0.17	24	12	Whole	NC
Total or mean	CPA: 13 cases; Interhemispheric cistern: 1 case	2.85	26	13.1	Whole: 7; Partial: 7	PR: 4; NC: 8; PG: 2

Target volumes of the radiosurgery ranged from 0.17 to 9.5 mm^3^ with a mean of 2.85 mm^3^. After the radiosurgery, the hyperactive cranial nerve symptoms improved relatively fast, sometimes dramatically. In several cases, the trigeminal neuralgia disappeared shortly after radiosurgery, sometimes within a month. In fact, most of the trigeminal neuralgia improved or totally disappeared. Moreover, functional control or recovery was maintained for a long time as shown in Table 3. The prompt relief of neuralgia was achieved in many cases. Facial spasms also improved in two cases. No further medications for neuralgia and facial spasm were not required in nine patients during 86 months after radiosurgery. In the majority of cases suffering from trigeminal neuralgia, the carbamazepine medication was discontinued shortly after radiosurgery in nine cases.

**Table 3:** Summary of the follow-up results for HCND after radiosurgery HCND: hyperactive cranial nerve dysfunction; Re: recurrence of trigeminal neuralgia; NC: no change

No	Age, years	Early response for HCND	Disappearance of HCND (months after radiosurgery)	Medication/last follow-up, months
1	42	6 months	Yes (12)	No/168
2	45	3 months	Yes (12)	No/168
3	39	7 months	Yes (7)	No/204
4	23	None	Diplopia	NC
5	43	1 month	Yes (1)	No/135
6	46	8 months	Yes (8)	No/15
7	65	8 months	Re	Re
8	80	1 month	Yes (12)	No/86
9	7	None	Hearing loss	NC
10	53	None	No deficit	NC
11	30	6 months	Yes (12)	Yes/12
12	66	1 month	Yes (1)	No/29
13	53	1 month	Yes (1)	No/20
14	69	1 month	Yes (1)	No/24
Total	-----	Yes: 11; None: 4	Yes (10)	No/9
Mean	47.2	-----	-----	Mean follow-up: 86.1 months

**Figure 4:**
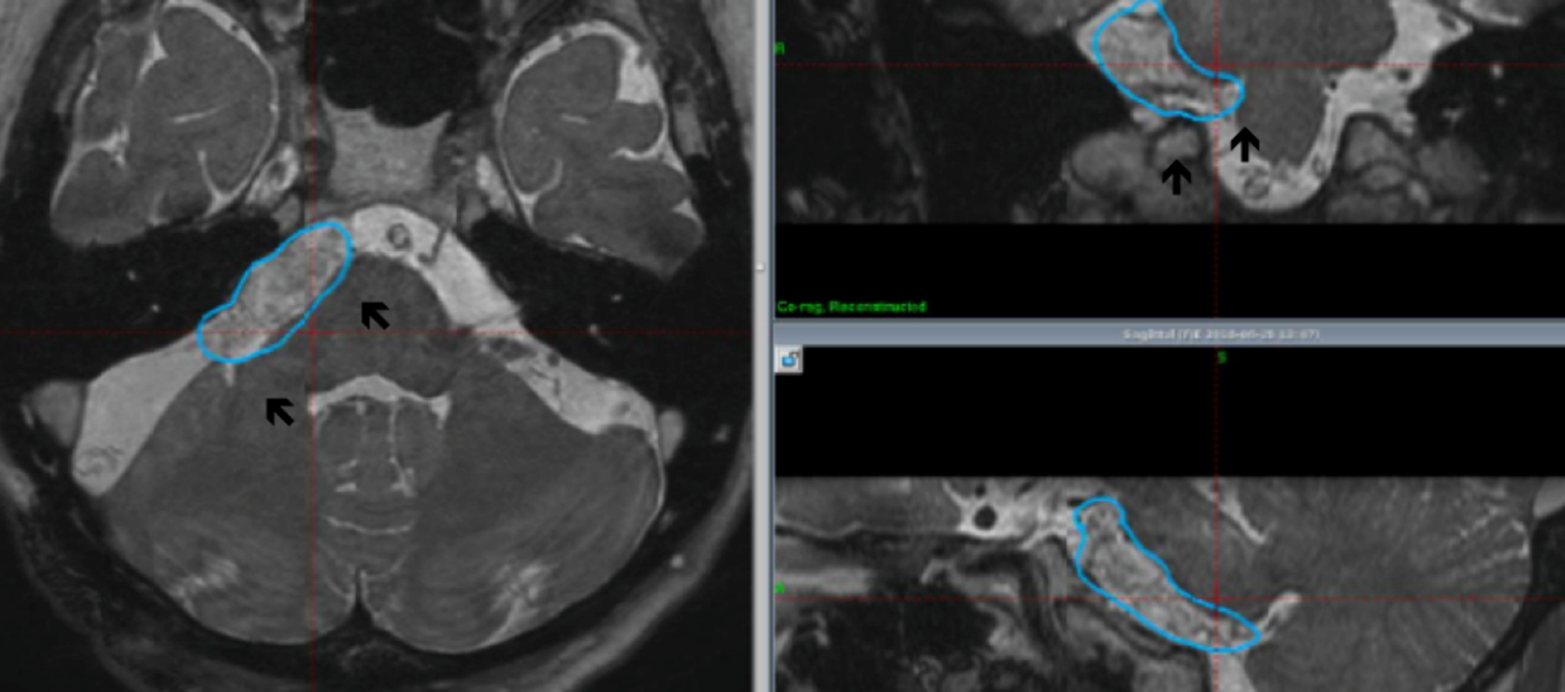
Whole tumor coverage (six months after radiosurgery) Apparent tumor shrinkage was observed (arrows) in some cases. The blue line indicates the tumor margin that was treated using gamma knife surgery

Tumor control was evaluated using serial MRI studies. In cases with whole tumor coverage, the tumor control was excellent, demonstrating a sufficient tumor shrinkage without any signs of tumor progression (Figure 4).

In cases with partial tumor coverage, only minor and partial tumor shrinkage was observed. However, because of tumor regrowth, subsequent tumor resection was required in two cases that had been treated with partial tumor coverage. Early relief from trigeminal neuralgia was achieved in seven cases. Facial spasms of the two cases also improved consistently within a few months and totally disappeared within one year after the procedure. No serious complications have occurred to date. Subsequent tumor resections were required in two cases because of tumor progression, but only in cases with partial tumor coverage. No perifocal edema associated with neurological signs was encountered.

## Discussion

Epidermoid tumors grow very slowly and have been reported to disappear spontaneously within several years. They often cause no neurological deficits even after developing into large tumors, chiefly in the CPA. However, they often become symptomatic because of cranial nerve involvements in the CPA or due to the direct compression of the cerebellum and brainstem. In fact, many cases suffer from HCND, including trigeminal or glossopharyngeal neuralgias as well as facial spasms [2,3]. The incidence of HCND is very high among cases of epidermoid tumors as the trigeminal or facial nerves are often involved and distorted with or without strangulation of the nerve inside the tumor. Abnormal compression or strangulation by the tumor is common and speculated to be the direct cause of such symptoms [4,5]. Spontaneous hemorrhage or chemical meningitis caused by the leakage of the cyst contents has been also reported in the past. These debilitating symptoms apparently may require some form of treatment. 

The mainstay of the treatment for this tumor is surgical tumor resection [4,5]. Despite the recent developments in microsurgery in and around the skull base, the surgical management of epidermoid tumors that are strongly attached to pia, arachnoid membrane or brainstem is extremely challenging because these tumors grow in close contact with cranial nerves and vascular structures that are often firmly attached to the arachnoid, cranial nerves or to brainstem. Extensive surgery may be associated with severe neurological sequelae, such as cranial nerve dysfunctions or brainstem damages.

Previous reports have shown that HCND, including trigeminal neuralgia and facial spasms, is more common in epidermoid tumors than in other CPA tumors such as meningiomas, schwannomas, or malignant skull base tumors [8,10,12]. Trigeminal nerves are often encased within an epidermoid tumor and the cholesterol materials that are produced by the tumor, and thus are more likely to be influenced or stimulated than in situations where only tumor compression is present. 

Prior to the advent of the operating microscope, the operative morbidity and mortality rates reportedly ranged from 20% to 57%. Although contemporary series have reported lower operative mortality rates, the morbidity rate for cranial nerves or brain structures continues to be an issue. Surgical removal of the tumor’s content is not difficult, but the resection of the tumor capsule, firmly attached to the brainstem or cranial nerves, is often harmful [4,5]. The rates of complete and incomplete resections have been inconsistent and the recurrence rates are not significantly different between these two groups. Serious adverse effects that impact the lower cranial nerves and brainstem can cause difficulty with swallowing and respiration.

Several clinical trials of standard fractionated radiotherapy for epidermoid tumors have been reported in the past with favorable results [13]. However, malignant transformation or the occurrence of squamous carcinomas has been reported after radiotherapy [14,15]. Radiosurgery trials for the management of epidermoid tumors have been reported in the literature (Table 4) [6,7,9]. Targets of radiosurgery were either tumor, nerve, or the interface. Successful improvement of trigeminal neuralgias and facial spasms was reported. Since these tumors have usually expanded widely over the surface of the brainstem and have often adhered to the surrounding brain tissue, radiosurgery can be difficult. 

**Table 4:** Current radiosurgical trials for the management of epidermoid tumors

Reporter	Cases	Method	Dose, Gy	Response	Remarks
1. Kida et al. [6]	7 (6 CPA)	Partial or total	14.6	No neuralgia (4/4)	Neuralgia disappeared; radiosurgical nerve decompression suspected
2. Cho KR et al. [8]	Meningioma: 30; schwannoma 18; Epidermoid: 1; AVM: 1	At fifth nerve root	90	BNI score improved from 3 to 2	Tumor size unchanged
3. El-Shehaby et al. [9]	12 cases (15 sessions)	Tumor fifth nerve root	11, 90	Neuralgia disappeared (5); facial spasm resolved (2/2)	Tumor size unchanged or shrank in 1
4. Vasquez JAJ et al. [7]	4 cases	Tumor fifth nerve	15, 80	Neuralgia improved	Good and safe alternative to microsurgery
5. Present series, Kida et al.	13 cases (14 sessions)	Tumor and nerve interface	13.1	Neuralgia facial spasm disappeared	Tumor control in 12/14 cases

In our treatment series, well-localized epidermoid tumors that were recognized using imaging studies responded well to radiosurgery. More importantly, prompt relief from trigeminal neuralgia and facial spasms were achieved using radiosurgery with a much lower radiation dose than that was required for the radiosurgery for classical trigeminal neuralgia. Moreover, the effects of pain control appeared very quickly after the procedure and were persistent and sustainable for a very long time. In fact, most of the cases did not require any further medication for neuralgia or facial spasms. Other CPA tumors, such as meningiomas and schwannomas, are sometimes associated with trigeminal neuralgia. The main cause of neuralgias appears to be the mass effects on the nerves, and the alleviation of such symptoms is often difficult without surgery.

In general, radiosurgery is known to have strong effects on the creation of intimal hypertrophy of arteriovenous malformation (AVM) vessels and the growth control of both benign and malignant brain tumors cells. Radiosurgery is believed to create a power of lesioning in the brain tissue to improve movement disorders and essential tremors. In the present series, radiosurgery for the treatment of epidermoid tumors utilized a different mechanism. First of all, trigeminal neuralgia can be specifically relieved using a low radiosurgical dose of less than 15 Gy at the margin; however, this dose would be insufficient to alleviate classical trigeminal neuralgia. Of importance, relief from neuralgia can be achieved very quickly after radiosurgery and can be sustained for a very long time as demonstrated in our series. Therefore, another kind of mechanism such as functional modulation may contribute to pain relief and the control of facial spasms. Mechanical decompression or the correction of nerve kinking might occur after radiosurgery-induced tumor shrinkage. However, such mechanisms were not apparent in our cases. Second, a decreased compression with offending vasculature around the nerve exit zone may happen. Again these mechanisms were not consistent with the findings in our cases. Finally, a decreased chemical stimulation with cholesterol materials or altered electrophysiological conditions at the nerve entry zone may be considered. The functional control for HCND, facial spasm, and trigeminal neuralgia is something unique as it works very quickly and consistently after the procedure. The theoretical basis of these effects has not yet been verified. The generation of abnormal discharges inside the nerve at the interface of the nerve and epidermoid tumor in the cistern may cause hyperactive cranial nerve dysfunction. Therefore, the irradiation of the interface using radiosurgery might alter this abnormal electrophysiological condition and stop the abnormal electrical discharges in the cranial nerves. 

We previously reported on the successful pain control of trigeminal neuralgia associated with epidermoid tumor in 2006 [6]. Since then, only a few reports on different radiosurgery methods have been published [7,9].

Future strategy for the management of epidermoid tumor

The mainstay of treatment for epidermoid cysts continues to be surgery. However, radiosurgical procedures seem to be very useful for the alleviation of HCND. If a tumor is small and well-localized, tumor control can also help to alleviate associated neuralgia symptoms. Thus, radiosurgery might be a useful tool for managing residual or recurrent tumors after surgery. More importantly, radiosurgery can be used to achieve functional control or functional modulation.

## Conclusions

Epidermoid tumors are particularly sensitive to radiosurgery. In fact, tumor control after the treatment is often favorable and tumor shrinkage can be achieved when the tumor is properly covered within the target volume. However, tumors with large volumes are difficult to cover completely. In conclusion, radiosurgery might be favored for epidermoid tumors when they are small and localized. Radiosurgery might also be suitable for residual tiny or thin-slice tumors or recurrent tumors after surgery. From a functional point of view, prompt relief from trigeminal neuralgia and other hyperactive nerve dysfunctions are especially beneficial to patients. Therefore, radiosurgery appears to be a very useful treatment for patients suffering from intractable trigeminal neuralgias and facial spasms associated with epidermoid tumors. 
